# Characteristics of patients with brain metastases from human epidermal growth factor receptor 2-positive breast cancer: subanalysis of Brain Metastases in Breast Cancer Registry

**DOI:** 10.1016/j.esmoop.2022.100495

**Published:** 2022-05-30

**Authors:** E. Laakmann, I. Witzel, T. Neunhöffer, T.-W. Park-Simon, R. Weide, K. Riecke, A. Polasik, M. Schmidt, J. Puppe, C. Mundhenke, K. Lübbe, T. Hesse, M. Thill, D.-M. Zahm, C. Denkert, T. Fehm, V. Nekljudova, J. Rey, S. Loibl, V. Müller

**Affiliations:** 1 Department of Gynecology, University Medical Center Hamburg-Eppendorf, Hamburg, Germany; 2 Frauenärzte am Dom, Mainz, HELIOS Dr. Horst Schmidt Kliniken Wiesbaden, Wiesbaden, Germany; 3 Medizinische Hochschule Hannover, Hannover, Germany; 4 Institut für Versorgungsforschung in der Onkologie, Koblenz, Germany; 5 Universitätsklinikum Ulm, Ulm, Germany; 6 Universitätsmedizin Mainz, Mainz, Germany; 7 Universitätsfrauenklinik Köln, Klinik und Poliklinik für Frauenheilkunde und Geburtshilfe, Köln, Germany; 8 Frauenklinik, Klinikum Bayreuth, Bayreuth, Germany; 9 Diakovere Henriettenstift, Breast Center, Hannover, Germany; 10 Agaplesion Diakonieklinikum Rotenburg, Rotenburg, Germany; 11 Agaplesion Markus Krankenhaus, Frankfurt, Germany; 12 Department of Gynecology, SRH Wald-Klinikum Gera GmbH, Gera, Germany; 13 Institut für Pathologie UKGM - Universitätsklinikum Marburg, Marburg, Germany; 14 Universitätsklinikum Düsseldorf, Düsseldorf, Germany; 15 GBG Forschungs GmbH, Germany

**Keywords:** brain metastases, breast cancer, HER2 positive, triple positive

## Abstract

**Background:**

Up to 40% of patients with metastatic human epidermal growth factor receptor 2 (HER2)-positive breast cancer develop brain metastases (BMs). Understanding of clinical features of these patients with HER2-positive breast cancer and BMs is vital.

**Patients and methods:**

A total of 2948 patients from the Brain Metastases in Breast Cancer registry were available for this analysis, of whom 1311 had primary tumors with the HER2-positive subtype.

**Results:**

Patients with HER2-positive breast cancer and BMs were—when compared with HER2-negative patients—slightly younger at the time of breast cancer and BM diagnosis, had a higher pathologic complete response rate after neoadjuvant chemotherapy and a higher tumor grade. Furthermore, extracranial metastases at the time of BM diagnosis were less common in HER2-positive patients, when compared with HER2-negative patients. HER2-positive patients had more often BMs in the posterior fossa, but less commonly leptomeningeal metastases. The median overall survival (OS) in all HER2-positive patients was 13.2 months (95% confidence interval 11.4-14.4). The following factors were associated with shorter OS (multivariate analysis): older age at BM diagnosis [≥60 versus <60 years: hazard ratio (HR) 1.63, *P* < 0.001], lower Eastern Cooperative Oncology Group status (2-4 versus 0-1: HR 1.59, *P* < 0.001), higher number of BMs (2-3 versus 1: HR 1.30, *P* = 0.082; ≥4 versus 1: HR 1.51, *P* = 0.004; global *P* = 0.015), BMs in the fossa anterior (HR 1.71, *P* < 0.001), leptomeningeal metastases (HR 1.63, *P* = 0.012), symptomatic BMs at diagnosis (HR 1.35, *P* = 0.033) and extracranial metastases at diagnosis of BMs (HR 1.43, *P* = 0.020). The application of targeted therapy after the BM diagnosis (HR 0.62, *P* < 0.001) was associated with longer OS. HER2-positive/hormone receptor-positive patients showed longer OS than HER2-positive/hormone receptor-negative patients (median 14.3 versus 10.9 months; HR 0.86, *P* = 0.03), but no differences in progression-free survival were seen between both groups.

**Conclusions:**

We identified factors associated with the prognosis of HER2-positive patients with BMs. Further research is needed to understand the factors determining the longer survival of HER2-positive/hormone receptor-positive patients.

## Highlights

• Patients with HER2-positive BMs from breast cancer have the best prognosis compared with other tumor subtypes.
• Among HER2-positive patients, hormone receptor-positive patients have the longest survival.
• HER2-targeted therapy is significantly associated with a better prognosis in patients with BMs.
• On average, two HER2-targeted therapy lines were administered prior to the development of BMs.
• New compounds are urgently needed to improve the outcome of this subgroup of patients.

## Introduction

The prognosis of patients with breast cancer brain metastases (BMs) is poor and could not be significantly improved within the last decades.^1^ In particular, patients with metastatic breast cancer and human epidermal growth factor receptor 2 (HER2)-positive or triple-negative tumor biology are at a higher risk for the development of BMs.^2^^,^^3^ A literature review reported incidence of 30%-55% in patients with metastatic HER2-positive breast cancer and 25%-46% in patients with metastatic triple-negative breast cancer (TNBC).^4^ An incidence of central nervous system metastases of 16.6% in patients with *de novo* metastatic breast cancer and 27.2% in patients with relapse metastatic breast cancer was reported in the Epidemiological Strategy and Medical Economics (ESME) registry.^5^ In addition, a systematic review by Koniali et al.^6^ identified young age, higher presenting stage, histological grade, tumor size, Ki-67 index and nodal involvement as independent risk factors of breast cancer BM development.

The prognosis of patients with BMs and breast cancer differs significantly between different tumor subtypes, with the highest survival rates for HER2-positive patients.^1^^,^^7^ Until now, only limited data were available regarding the specific characteristics of patients with BMs of HER2-positive breast cancer.

The aim of this analysis was to characterize a large, real-world cohort of HER2-positive patients with BMs, to compare their clinical characteristics with those of other tumor subtypes, as well as to evaluate the survival rates and factors associated with survival in HER2-positive patients with BMs. Furthermore, we analyzed the therapeutic modalities for patients with BMs of an HER2-positive breast cancer. The clinical data for the evaluation were derived from our large Brain Metastases in Breast Cancer (BMBC) registry: a multicenter German registry in which the clinical data of patients with BMs of breast cancer are documented.

Analysis of factors associated with survival in HER2-positive patients with BMs could help to optimize the diagnostic and therapeutic approaches for this patient cohort. Furthermore, we intend to initiate a scientific database for future research projects that could help to further clarify the mechanisms of BM development in HER2-positive patients.

## Patients and methods

Patient clinical data from the BMBC registry were analyzed. The BMBC registry is a German multicenter clinical database. The clinical data of patients diagnosed with BMs from breast cancer from the year 2000 onward has—prospectively and retrospectively—been collected in the registry. Patient data registered before 5 December 2020 were included in this analyses. A data snapshot from 4 January 2021 was taken after data cleaning. The aims of this evaluation were

1a. To characterize the cohort of HER2-positive patients (HER2 positive in the primary breast cancer histology) with breast cancer BMs and to compare the clinical characteristics of this cohort with patients with TNBC or luminal-like breast cancer. Luminal-like is defined as estrogen receptor (ER) and/or progesterone receptor (PgR) positive and HER2 negative. In this study, luminal-A- and B-like patients were treated as one collective cohort.
1b. To characterize the cohort of HER2-positive/hormone receptor-positive (ER and/or PgR receptor positive) patients with breast cancer BMs and to compare the clinical characteristics of this cohort with patients with HER2-positive/hormone receptor-negative (ER and PgR receptor-negative) breast cancer.
2. To estimate and compare the overall survival (OS) and progression-free survival (PFS) after the diagnosis of BMs, including the brain PFS and extracranial PFS, in different breast cancer subtypes.

For the endpoints OS and PFS, univariate and multivariate Cox regression analyses were carried out in the subgroup of HER2-positive patients, adjusting for the following covariates: age, hormone receptor status, performance status according to the Eastern Cooperative Oncology Group (ECOG) status, number, maximum size and localization of BMs, clinical signs of BMs, extracranial metastases at the time of BM diagnosis, progress of extracranial metastases in the further course of the disease, localization of first extracranial metastases and application of systemic therapy.

Continuous data were summarized using total number, mean, standard deviation, median, minimum and maximum for each group. Categorical and ordinal data were summarized using the number and percentage of patients in each group. OS was defined as the time interval between first diagnosis of BMs and death due to any reason. PFS was defined as the time interval between first diagnosis of BMs and extracranial progress (including the first occurrence of extracranial metastases from 60 days after diagnosis of BMs), or BM progress or death. For these endpoints, as well as for the time between breast cancer diagnosis and BM diagnosis (in patients developing extracranial metastases after the diagnosis of BMs) and for the time between breast cancer diagnosis and extracranial metastases diagnosis (in patients developing BMs after the diagnosis of extracranial metastases), Kaplan–Meier curves; the median survival times and the survival rates after 1, 2, 3 and 4 years, with the corresponding 95% confidence intervals (CIs), were determined. Differences in the survival curves were tested by the log-rank test. All reported *P*-values were two sided, and the significance level was set to 0.05. CIs symmetrically cover 95%. Adjustment for multiple testing was not planned. The data were analyzed using SAS (Statistical Analysis Software, Cary, NC,) version 9.4 with SAS Enterprise Guide Version 7.1 on Microsoft Windows 10 Enterprise (Microsoft, Redmond, WA).

### Ethics approval

Ethical approval for this study was obtained from Ethikkommission bei der Landesärztekammer Hessen (approval number: FF42/2013).

## Results

### Patients’ characteristics

Clinical data from 2948 patients in the BMBC registry were available for analysis. About 44% (*n* = 1311) of patients had an HER2-positive tumor biology, while 56% (*n* = 1637) of patients’ tumors were HER2 negative. Among the HER2-negative tumors, 33% (*n* = 976) were luminal like and 22% (*n* = 661) were triple negative.

In the following, the characteristics of patients with HER2-positive tumor biology will be analyzed and compared with those of patients with HER2-negative tumors.

Among the HER2-positive patients with BMs, the median age of first breast cancer diagnosis was 52 years. Approximately one-third of these patients were diagnosed with breast cancer between 2010 and 2014 (*n* = 399, 31%), one-third between 2005 and 2009 (*n* = 362, 28%) and 21% (*n* =  273) between 2000 and 2004.

About 58% (*n* = 731) of primary tumors were ER and/or PgR positive, while 42% (*n* = 528) both ER and PgR negative. Information on the hormone receptor status was missing for 52 patients (4%). Nearly 60% of the primary tumors were poorly differentiated (G3; *n* = 713). A molecular subtype switch to HER2 negative was observed in 2.3% (*n* = 30) of the originally HER2-positive tumors in a follow-up biopsy. Most patients (74%, *n* = 566) had an initial breast cancer tumor size <5 cm. Roughly 42% (*n* = 352) were treated with neoadjuvant chemotherapy, the majority of which did not achieve pathologic complete response (pCR; 79%, *n* = 1224). The median age at diagnosis of extracranial metastases was 55 years (range 22-93 years). As many as 829 patients were diagnosed with extracranial metastases before BM diagnosis (63%). The median time from breast cancer to BM diagnosis was 19 months (in the cohort of patients with extracranial metastases after BM diagnosis; range 17-21 months). At the time of BM diagnosis, the median age was 56 years (range 22-93 years). More than half of the patients with known ECOG status were in a good general condition (64%, *n* = 374 with ECOG 0 and 1) at the time of BM diagnosis. Most patients had extracranial metastases at the time of BM diagnosis (78%, *n* = 1018). The most frequent localizations of extracranial metastases at the time of BM diagnosis were bone, liver and lung (*n* = 525, 40%; *n* = 397, 30% and *n* = 375, 29%, respectively). The most common localization at the time of first BM diagnosis in HER2-positive patients was fossa anterior (63%, *n* = 821), whereas 58% (*n* = 759) showed metastases in the fossa posterior. Leptomeningeal metastases was rare, as the meninges were affected in only 9% patients. Concerning the number of BMs, 30% of patients (*n* = 363) had one BM at the time of first diagnosis, 27% (*n* = 330) had two to three BMs and 44% (*n* = 538) had four or more BMs. Most of the patients (77%, *n* = 1009) had neurological symptoms at the time of BM diagnosis, while 23% (*n* = 302) were asymptomatic.

Compared with other subtypes, HER2-positive patients with BMs were slightly younger at the time of breast cancer (median of 52 years for HER2 positive versus 53.0 for HER2 negative; *P* < 0.001) and BM diagnosis (median age 56 years for HER2-positive versus 58 for HER2-negative; *P* = 0.009). HER2-positive patients had a higher pCR rate after neoadjuvant chemotherapy (22% for HER2 positive versus 12% for HER2 negative; *P* = 0.002) and slightly more G3 tumors (60% for HER2 positive versus 57% for HER2 negative; *P* < 0.001). Differences among the breast cancer subtypes could also be detected in the evaluation of the BM patterns: HER2-positive patients had more often BMs in the posterior fossa (58% versus 49%; *P* < 0.001) and less often leptomeningeal disease (9% versus 19%; *P* < 0.001). Patients with HER2-positive breast cancer had a slightly lower rate of extracranial metastases at the time of BM diagnosis (78% versus 80%; *P* < 0.001). The detailed analysis showed a higher rate of liver metastases (30% versus 22%; *P* = 0.005) and a lower rate of lung metastases (29% versus 33%; *P* < 0.001) as the first extracranial metastases localization in HER2-positive patients. Detailed patient characteristics are shown in Table 1.

**Table 1:** Characteristics of patients with breast cancer and BM according to subtype

Parameter	All patients *n* = 2948	HER2-positive *n* = 1311	HER2-negative *n* = 1637	Luminal like *n* = 976	TNBC *n* = 661	*P*-value^a^
Age at the time of first breast cancer diagnosis^b^ (years), median (range)	52.0 (20.0-98.0)	52.0 (21.0-92.0)	53.0 (20.0-98.0)	55.0 (20.0-91.0)	51.0 (25.0-98.0)	<0.001
Age at the time of BM diagnosis^b^ (years), median (range)	57.0 (22.0-99.0)	56.0 (22.0-93.0)	58.0 (25.0-99.0)	60.0 (25.0-91.0)	54.0 (27.0-99.0)	0.009
pCR rate, *n* (%)	153 (16.2)	87 (21.5)	66 (12.2)	18 (6.9)	48 (17.1)	0.002
Tumor grade: G3, *n* (%)	1577 (57.9)	713 (59.5)	864 (56.5)	395 (43.2)	469 (76.4)	<0.001
Localization of BMs: posterior fossa, *n* (%)	1561 (53.0)	759 (57.9)	802 (49.0)	443 (45.4)	359 (54.3)	<0.001
Leptomeningeal metastases, *n* (%)	435 (14.8)	122 (9.3)	313 (19.1)	218 (22.3)	95 (14.4)	<.001
Extracranial metastases at the time of BM diagnosis, *n* (%)	2328 (79.0)	1018 (77.7)	1310 (80.0)	821 (84.1)	489 (74.0)	<.001
Bone metastases as the first extracranial metastases, *n* (%)	1179 (40.0)	525 (40.1)	654 (40.0)	513 (52.7)	141 (21.3)	<0.001
Liver metastases as the first extracranial metastases, *n* (%)	763 (25.9)	397 (30.3)	366 (22.4)	241 (24.7)	125 (18.9)	0.005
Lung metastases as the first extracranial metastases, *n* (%)	921 (31.3)	375 (28.6)	546 (33.4)	275 (28.2)	271 (41.0)	<0.001
Skin metastases as the first extracranial metastases, *n* (%)	130 (4.4)	49 (3.7)	81 (5.0)	37(3.8)	44(6.7)	0.010

BM, brain metastasis; HER2, human epidermal growth factor receptor 2; pCR, pathologic complete response; TNBC, triple-negative breast cancer.

a Fisher’s exact test respectively. Chi-square test between HER2-positive and HER2-negative patients.

b Information on age at diagnosis of breast cancer and BMs was missing for one HER2-positive patient.

**Figure 1:**
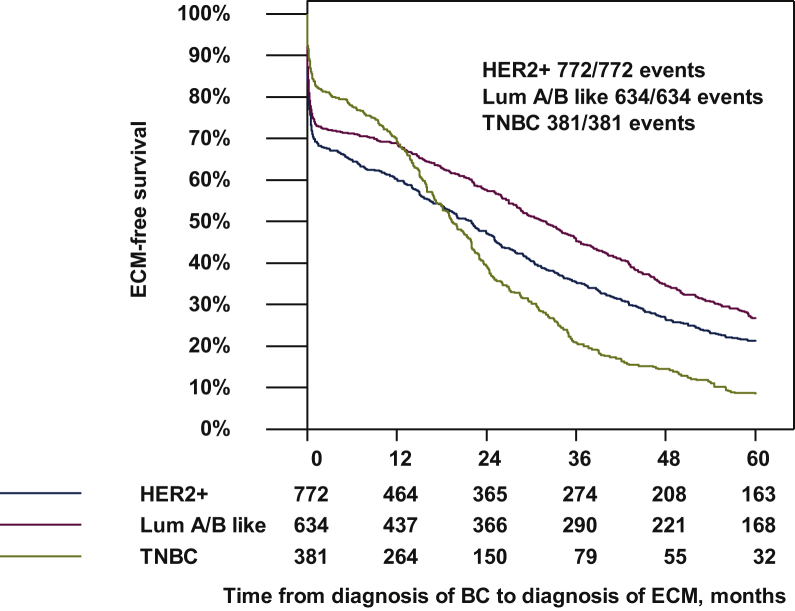
**Time from diagnosis of breast cancer (BC) to diagnosis of extracranial metastases.** ECM, extracranial metastases; Lum A/B like, luminal-A- and B-like; TNBC, triple-negative breast cancer.

In addition, we carried out an analysis of time from breast cancer diagnosis to extracranial metastases in different tumor subtypes for patients that developed BMs after the diagnosis of extracranial metastases (Figure 1). Statistically significant differences could be observed between the groups. The estimated 1-year extracranial metastases-free survival was significantly lower for HER2-positive patients compared with luminal-like or TNBC patients (60%, 95% CI 57%-64% versus 69%, 95% CI 65%-72% versus 69%, 95% CI 64%-73%, respectively; Figure 1). In the follow-up period, a lower extracranial metastases-free survival rate could be observed, especially for TNBC: the estimated 2-year extracranial metastases-free survival for TNBC was 39% (CI 95% 35%-44%), 47% for HER2-positive (95% CI 44%-51%) and 58% for luminal-like patients (CI 95% 54%-62%).

Among HER2-positive patients, HER2-positive/hormone receptor-positive (triple-positive) patients had different clinical characteristic compared with HER2-positive/hormone receptor-negative patients. The statistical analysis indicated that triple-positive patients were slightly younger at the time of breast cancer diagnosis (median 50 years versus 53 years; *P* = 0.023), had a smaller tumor size (breast cancer <5 cm 78% versus 67%; *P* = 0.003) and a lower rate of differentiation of the primary tumor (G3 tumor differentiation in 53% versus 70%; *P* < 0.001) than HER2-positive/hormone receptor-negative patients. Regarding the metastatic patterns, triple-positive patients had a higher rate of extracranial metastases at the time of BM diagnosis (80% versus 75%; *P* = 0.05) and a higher rate of leptomeningeal disease (11% versus 7%; *P* = 0.01). Furthermore, triple-positive patients were significantly more often neurologically asymptomatic at the time of BM diagnosis (25% versus 18%; *P* = 0.002). Detailed characteristics of HER2-positive patients according to hormone receptor status are described in Table 2.

**Table 2:** Characteristics of HER2-positive patients with breast cancer and BMs according to hormone receptor status

Parameter	HER2 positive overall (*n* = 1259)	HER2 positive/hormone receptor negative (*n* = 528)	HER2 positive/hormone receptor positive (*n* = 731)	*P*-value
Age at the time of breast cancer first diagnosis (years), median (range)^a^	52.0 (21.0-92.0)	53.0 (21.0-85.0)	50.0 (23.0-92.0)	0.023
Initial breast cancer tumor size (pT1 + pT2), *n* (%)	543 (73.6)	189 (67.0)	354 (77.8)	0.003
Tumor grade: G3, *n* (%)	703 (59.9)	339 (70.0)	364 (52.8)	<0.001
Leptomeningeal metastases, *n* (%)	117 (9.3)	36 (6.8)	81 (11.1)	0.010
Extracranial metastases at the time of BM diagnosis, *n* (%)	979 (77.8)	396 (75.0)	583 (79.8)	0.047
Bone metastases as the first extracranial metastases, *n* (%)	503 (40.0)	164 (31.1)	339 (46.4)	<0.001
Neurological symptoms at the time of BM diagnosis, *n* (%)	979 (77.8)	433 (82.0)	546 (74.7)	0.002

BM, brain metastasis; HER2, human epidermal growth factor receptor 2.

a Information on age at diagnosis of breast cancer and BM was missing for one HER2-positive patient.

### Survival analysis in the overall HER2-positive cohort

**Figure 2:**
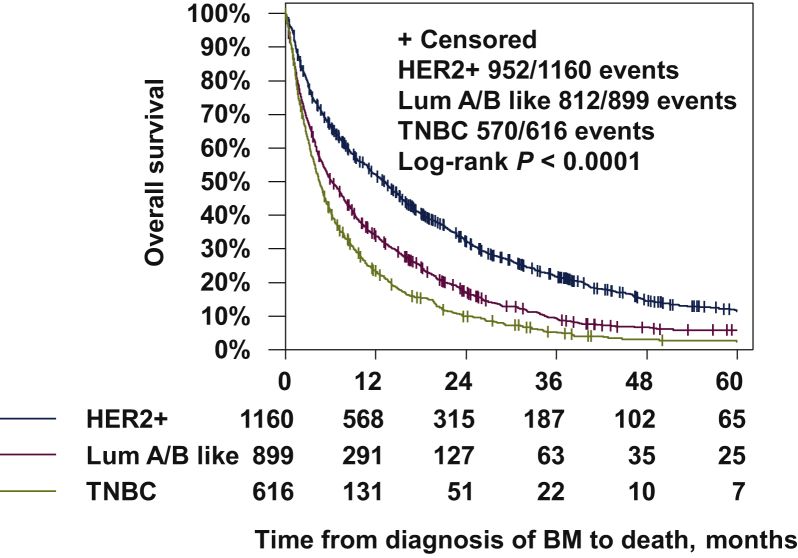
**Overall survival (OS) of patients with brain metastases (BMs) in different breast cancer subtypes.** Lum A/B like, luminal-A- and B-like; TNBC, triple-negative breast cancer.

The median OS in the cohort of 2675 patients with BMs in the BMBC registry was 7.7 months (95% CI 7.1-8.2). In the statistical analysis, a significantly higher OS rate could be identified for HER2-positive patients, compared with luminal like or TNBC (median OS 13 versus 6 versus 5 months and 95% CI 11-14 versus 5-7 versus 4-5 months, respectively; *P* < 0.0001; Figure 2).

In univariate analysis, older patients (≥60 years) had a lower probability to survive compared with patients <60 years [hazard ratio (HR) 1.63, 95% CI 1.43-1.86]. Furthermore, patients with hormone receptor-negative status (HR 0.86, 95% CI 0.76-0.98); lower performance status (HR 1.97, 95% CI 1.61-2.40); higher number of BMs (2-3 versus 1: HR 1.50 95% CI 1.25-1.80; ≥4 versus 1: HR 1.82 95% CI 1.54-2.14); no application of chemotherapy, endocrine therapy, or targeted therapy after BM diagnosis (HR 0.63, 95% CI 0.56%-0.72%; HR 0.54 95% CI 0.44-0.67; and HR 0.54 with 95% CI 0.47-0.61, respectively) had a significantly worse OS. BMs in the fossa anterior (HR 1.23, 95% CI 1.07-1.40), leptomeningeal disease, (HR 1.50 with 95% CI 1.21-1.87), neurological symptoms at BM diagnosis (HR 1.26 with 95% CI 1.08-1.47) and extracranial metastases at BM diagnosis (HR 1.69 with 95% CI 1.44-2.00) or in the further course of the disease were associated with a shorter OS. No significant difference could be observed in OS for patients with late versus early onset of BMs (data not shown).

**Figure 3:**
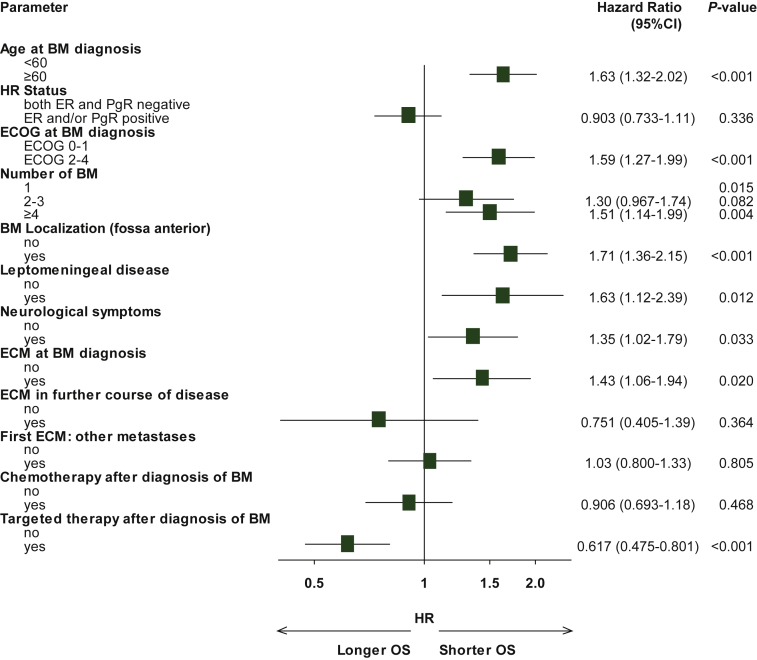
**Multivariate analysis of factors associated with overall survival (OS) in HER2-positive patients with brain metastases (BMs).**ECM, extracranial metastases; ECOG, Eastern Cooperative Oncology Group; ER, estrogen receptor; HER2, human epidermal growth factor receptor 2; HR, hormone receptor; OS, overall survival; PgR, progesterone receptor.

In multivariate analysis, the following factors were significantly associated with a shorter OS in HER2-positive patients: higher age (>60 versus ≤60: HR 1.63, 95% CI 1.32-2.02), low performance status (ECOG 2-4 versus ECOG 0-1: HR 1.59, 95% CI 1.27-1.99), higher number of BMs (2-3 versus 1: HR 1.30 95% CI 0.97-1.74; ≥4 versus 1: HR 1.51 with 95% CI 1.14-1.99), localization of BMs in the fossa anterior (yes versus no: HR 1.71, 95% CI 1.36-2.15), or leptomeningeal disease (yes versus no: HR 1.63, 95% CI 1.12-2.39), neurological symptoms at BM diagnosis (yes versus no: HR 1.35, 95% CI 1.02-1.79), as well as extracranial metastases at the time of BM diagnosis (yes versus no: 1.43, 95% CI 1.06-1.94). Application of HER2-targeted therapy after the BM diagnosis was significantly associated with a longer OS (yes versus no: HR 0.62, 95% CI 0.48-0.80). Detailed information is shown in Figure 3.

A median PFS of 5.4 months (95% CI 5.0-5.7) after the diagnosis of BMs could be calculated for the overall cohort of patients with BMs. Patients with HER2-positive breast cancer had a significantly longer PFS compared with luminal like or TNBC (median PFS 7.3 versus 4.5 versus 3.5 months, 95% CI 6.5-7.9 versus 4.0-5.1 versus 3.2-4.0 months, respectively; *P* < 0.0001; Supplementary Figure S1, available at https://doi.org/10.1016/j.esmoop.2022.100495).

In a multivariate analysis, higher age (≥60 years), lower performance status, higher BM number, BM localization in fossa anterior, neurological symptoms at BM diagnosis and extracranial metastases at BM diagnosis were associated with a significantly lower PFS. HER2-targeted therapy was the only factor significantly associated with a better PFS in HER2-positive patients with BMs (Supplementary Figure S2, available at https://doi.org/10.1016/j.esmoop.2022.100495).

Most patients (*n* = 780, 93.6% of HER2-positive and *n* = 1175, 95.3% of HER2-negative) had a tumor-related cause of death. Approximately one-third of patients in the HER2-positive and HER2-negative cohorts died due to BM alone. Approximately one-quarter of patients died due to BMs and extracranial metastases (detailed information in Supplementary Table S1, available at https://doi.org/10.1016/j.esmoop.2022.100495).

### Survival analysis of HER2-positive patients according to hormone receptor status

Furthermore, we carried out an analysis of survival of HER2-positive patients according to hormone receptor status. A significantly longer OS was detected for patients with HER2-positive/hormone receptor-positive subtype versus HER2-positive/hormone receptor-negative subtype (median 14.3, 95% CI 12.4-15.9 versus median 10.9, 95% CI 9.2-12.7; Supplementary Figure S3, available at https://doi.org/10.1016/j.esmoop.2022.100495).

The univariate analysis of factors associated with OS in HER2-positive/hormone receptor-positive patients showed that higher age (≥60 years: HR 1.83, 95% CI 1.53-2.17), lower performance status (HR 1.90, 95% CI 1.45-2.59), higher number of BMs (2-3 versus 1: HR 1.52 95% CI 1.19-1.94; ≥4 versus 1: HR 1.91, 95% CI 1.53-2.38), localization in fossa anterior (HR 1.21, 95% CI 1.01-1.44), leptomeningeal metastases (HR 1.49, 95% CI 1.14-1.96), neurological symptoms at BM diagnosis (HR 1.21, 95% CI 0.99-1.47) and extracranial metastases at BM diagnosis (HR 1.71, 95% CI 1.35-2.15) or in the further course of the disease were significantly associated with a shorter OS. Chemotherapy, endocrine therapy and/or targeted therapy after diagnosis of BMs were significantly associated with a longer OS (HR 0.625, 95% CI 0.52-0.74; HR 0.52, 95% CI 0.41-0.64 and HR 0.55, 95% CI 0.46-0.66, respectively).

Among patients with extracranial metastases before the BM diagnosis, patients with an HER2-positive/hormone receptor-positive tumor biology had a significantly longer time period to development of extracranial metastases: the estimated 4-year extracranial metastases-free survival in HER2-positive/hormone receptor-negative patients was 16.2% (95% CI 12.2%-20.6%) versus 31.2% (95% CI 27.0%-35.5%) in HER2-positive/hormone receptor-positive patients.

No significant difference could be observed concerning OS in HER2-positive/HR-positive patients when stratifying for early or late onset of BMs.

In a multivariate analysis, higher age (≥60 years), lower performance status, higher number of BMs, BM localization in fossa anterior and extracranial metastases at BM diagnosis were significantly associated with a shorter OS. Endocrine and targeted therapy were significantly associated with a better OS in HER2-positive/hormone receptor-positive patients (Supplementary Figure S4, available at https://doi.org/10.1016/j.esmoop.2022.100495).

There was no significant difference in PFS between HER2-positive/hormone receptor-positive and HER2-positive/hormone receptor-negative patients (7 months in both groups).

### Therapeutic modalities of HER2-positive patients with BMs

In total, clinical data from 2428 patients were available for analysis. Concerning the local treatment of BMs, two-thirds of the patients (66%, *n* = 758) were treated with brain radiotherapy alone. Among them, most received whole brain radiation therapy (WBRT; *n* = 611, 81%). Approximately one-third of patients with BMs (28%, *n* = 325) were treated with a combination of surgery and radiotherapy, in which the most common regimen was a combination of surgery and WBRT; 62% of patients (*n* = 202) were treated with this modality (Supplementary Table S2, available at https://doi.org/10.1016/j.esmoop.2022.100495).

Clinical data from 1257 patients were available for the evaluation of systemic treatment regimens—for both the primary tumor and metastatic disease—in HER2-positive patients with BMs. Around 86% (*n* = 1127) of patients were treated with a chemotherapy before and 40% (*n* = 519) after the BM diagnosis. Endocrine therapy was applied in 63% (*n* = 459) of the hormone receptor-positive patients before the BM diagnosis and in 18% (*n* = 134) after the BM diagnosis. Approximately 70% (*n* = 917) of the patients were treated with HER2-targeted therapy before the BM diagnosis. The most prescribed compound was trastuzumab: 88% (*n* = 804) of patients were treated with this HER2-targeted agent. Nearly 24% (*n* = 218) of patients were treated with a combination of trastuzumab and pertuzumab, 12% (*n* = 114) were treated with lapatinib and 7% (*n* = 61) of patients were treated with T-DM1. Among the 917 patients who underwent an HER2-targeted therapy before the BM diagnosis, 1513 HER2-targeted therapy lines were applied (*n* = 365 adjuvant, *n* = 189 neoadjuvant, *n* = 958 metastatic, *n* = 1 missing data).

On average, two HER2-targeted therapy lines (mean 1.7) were applied during the breast cancer disease but before the occurrence of BMs.

After the BM diagnosis, 37% (*n* = 486) of patients were treated with HER2-targeted therapy: 50% (*n* = 243) with trastuzumab, 48% (*n* = 234) with lapatinib, 32% (*n* = 157) with trastuzumab-emtansin-1 and 16% (*n* = 76) with a combination of trastuzumab and pertuzumab. The 486 aforesaid patients were treated with anti-HER2-targeted therapies in a total of 828 therapy lines (for one patient in one therapy line the setting was not specified). On average, two HER2-targeted therapy lines were applied after the diagnosis of BMs.

## Discussion

Our analyses of 2948 patients with BMs of breast cancer (including 1311 patients with an HER2-positive subtype) showed that patients with HER2-positive BMs of breast cancer have the best prognosis compared with other tumor subtypes. Factors significantly associated with the prognosis of HER2-positive patients with BMs were age, performance status, number and localization of BMs, neurological symptoms at the time of BM diagnosis, extracranial metastases at the time of BM diagnosis and application of HER2-targeted therapy after the BM diagnosis.

Our results are in line with the analyses from other patient cohorts. Darlix et al.^5^ evaluated 1027 patients with HER2-positive breast cancer and BMs. In a multivariable analysis, they found that older age, hormone receptor negativity, a higher number of metastatic sites and no administration of a previous HER2-targeted therapy were prognostic risk factors associated with a shorter OS. Furthermore, they described that a higher number of previous chemotherapy lines was associated with a shorter OS.^5^ Morikawa et al.^8^^,^^9^ evaluated the clinical characteristics of 100 patients with HER2-positive breast cancer with BMs who underwent radiation therapy as the primary BM treatment. Significantly better survival was reported for patients with higher performance status, a lower number of BMs, continued use of HER2-targeted therapy after BM diagnosis and better control of extracranial disease. An absence of neurological symptoms at the time of BM diagnosis was significantly associated with longer OS in univariate and multivariate analyses. Gori et al.^10^ evaluated a cohort of 154 patients with BMs of HER2-positive breast cancer. They identified that patients with BMs of HER2-positive breast cancer treated locally with surgery/stereotactic radiosurgery and systemically with HER2-targeted therapy experienced the better outcomes. Masci et al.^11^ evaluated 109 patients with BMs of HER2-positive breast cancer. In this cohort, prognostic factors identified were number of central nervous system metastases, brain irradiation and implementation of HER2-targeting therapies.

Furthermore, among HER2-positive patients, we identified a group with distinguishing clinical characteristics: patients with an HER2-positive/hormone receptor-positive tumor biology. To our knowledge, we evaluated the largest cohort of triple-positive patients with BMs to date. In these 731 triple-positive patients analyzed, HER2-positive/hormone receptor-positive patients—when compared with HER2-positive/hormone receptor-negative patients—were slightly younger at the time of breast cancer diagnosis, had a smaller breast cancer tumor size and a lower rate of low primary tumor differentiation. Furthermore, HER2-positive/hormone receptor-positive patients had a higher rate of extracranial metastases at the time of BM diagnosis and a higher rate of leptomeningeal metastases (11% versus 7%) and were significantly more often neurologically asymptomatic at the time of BM diagnosis. A significantly better OS was observed for triple-positive patients compared with HER2-positive/hormone receptor-negative patients. Remarkably, a better OS could be seen despite a higher rate of extracranial metastases and leptomeningeal metastases, factors which are generally associated with a poor survival in patients with BMs.^8^^,^^12^, ^13^, ^14^ A possible reason for the higher OS in this cohort could be the option of endocrine therapy in addition to an HER2-targeted therapy. Regarding this hypothesis, the potential improvement of the standard endocrine therapy with, for example, cyclin-dependent kinase 4 and 6 (CDK4/6) inhibitors should be examined in clinical trials for HER2-positive patients with BMs. Another possible explanation for the better OS of triple-positive patients with BMs could be a different tumor biology of triple-positive breast cancer. Lekanidi et al.^15^ evaluated 60 patients with BMs of HER2-positive breast cancer according to hormone receptor status. HER2-positive/ER-negative women were more likely to present with a larger number of lesions, more brain stem/occipital metastases and hydrocephalus. The authors concluded that these characteristics may predispose hormone receptor-negative patients to unfavorable outcomes following treatment.

The PFS of HER2-positive/hormone receptor-positive and HER2-positive/hormone receptor-negative patients did not differ in our cohort. Pasquier et al.^16^ evaluated the neurological PFS in the ESME registry. In contrast to our results, Pasquier et al.^16^ showed a higher neurological PFS for HER2-positive/hormone receptor-positive versus HER2-positive/hormone receptor-negative patients (8.8 months versus 6.9 months, respectively). A possible explanation for this disparity is that, in our evaluation, PFS was defined by extracranial and/or intracranial progression. Pasquier et al.^16^ put the focus on the neurological progression alone.

Our evaluation showed that the absence of neurological symptoms at the time of BM diagnosis is significantly associated with a better OS and PFS. The retrospective, noninterventional, nonrandomized design of our evaluation does not allow for the assumption of a causal relationship between these aspects. A recently published analysis by Laakmann et al.^17^ showed a trend that asymptomatic patients have a less severe metastatic disease in the brain and, despite a less intensive local BM therapy, have nonetheless better outcomes (statistically significant for the cohort of HER2-positive patients) than patients who present with symptomatic BMs. However, a lead time bias due to an earlier diagnosis cannot be ruled out. In summary, these observations emphasize the need for trials to examine the benefit of early detection and treatment of BMs in patients with breast cancer. Three studies are currently recruiting patients for the investigation of magnetic resonance screening of BMs in patients with breast cancer (NCT04030507, NCT03881605^18^ and NCT03617341).

Concerning the local treatment of BMs, most patients were treated with a brain radiotherapy alone. Among them, the majority (81%) received WBRT. The high rate of the WBRT as a stand-alone local treatment is probably due to the retrospective design of the data collection. Furthermore, the retrospective design of our analysis possibly explains the low pCR rate (21.5%) of HER2-positive patients in our cohort, in comparison to currently known pCR rates.^19^

Regarding systemic treatment, all subgroup analyses showed that HER2-targeted therapy after BM diagnosis is significantly associated with a better prognosis. Because of the retrospective design of the presented analyses, only a few patients were treated with novel HER2-targeted agents, such as trastuzumab deruxtecan, tucatinib or neratinib. According to recent knowledge, some evidence suggests that HER2-targeted substances can pass the blood–brain barrier. Kabraji et al.^20^ summarized data from radiolabeled imaging and clinical responses in patients, coupled with antibody-binding analyses and downstream target inhibition in orthotopic breast cancer BMs. Their results strongly indicate HER2 antibody penetration across the blood–tumor barrier in existing metastases.^20^ For trastuzumab, Terrell-Hall et al.^21^ demonstrated that, although in small and most likely not efficacious quantities, trastuzumab does cross the blood–brain and blood–tumor barriers. A disrupted blood–brain barrier potentially has an impact on the permeability for HER2-targeted substances.^22^^,^^23^ A further possible explanation for the prolonged survival under HER2-targeted therapy is the controlled extracranial disease.

Until recently, only a limited number of HER2-directed therapies were implemented. Our analyses detected that, on average, two HER2-targeted therapy lines were already applied before the development of BMs. This indicates that new HER2-targeted compounds, which can achieve a therapeutically relevant concentration in the central nervous system, are urgently needed to improve the outcome of this subgroup of patients. As such, new therapeutical developments such as tucatinib,^24^ neratinib^25^ or trastuzumab deruxtecan^26^ are likely to further improve patient outcomes.^27^ A recently published review from Simmons et al.^28^ underlines and summarizes the positive impact of modern HER2-targeted substances on PFS and OS of patients with breast cancer.^28^ Garcia-Alvarez et al.^29^ carried out a literature review on currently available novel HER2-targeted substances influencing BMs in patients with breast cancer and summarized that, aside from anti-HER2 monoclonal antibodies, tyrosine kinase inhibitors and antibody–drug conjugates have exhibited antineoplastic intracranial activity, either by affecting established central nervous system metastases or by delaying the time until development of subsequent BMs. Furthermore, the importance of a multidisciplinary approach was recently described by Stavrou et al.^30^

### Conclusions

In conclusion, analyses of this large cohort demonstrated that patients with HER2-positive BMs of breast cancer have the best prognosis, when compared with other tumor subtypes. Among HER2-positive patients, hormone receptor-positive patients have the longest OS. HER2-targeted therapy is significantly associated with a better prognosis in all subgroups of patients with BMs of breast cancer. Until recently, only a limited number of HER2-directed therapies were being implemented and 70% of patients had an anti-HER2 targeted therapy before development of BMs. New compounds and treatment strategies are urgently needed to improve the outcome of this patient subgroup. Additional research should be carried out to further understand the factors associated with the better prognosis of the triple-positive breast cancer patients with BMs.
