# Supplementary material for: Characteristics of patients with brain metastases from human epidermal growth factor receptor 2-positive breast cancer: subanalysis of Brain Metastases in Breast Cancer Registry
Source: ESMO Open. 2022 May 30;7(3):100495. doi: 10.1016/j.esmoop.2022.100495 (PMC9271494; doi:10.1016/j.esmoop.2022.100495)
Supplement: Supplementary Tables S1 and S2 [file mmc1.docx]

Supplementary Table S1: Causes of death in HER2-negative and HER2-positive patients (supplementary material)

| **Parameter** | **Category** | **HER2-positive**  **n=1311**  **(%)** | **HER2-negative**  **n=1637**  **(%)** | **Overall**  **n=2948**  **(%)** | **p-value** |
| --- | --- | --- | --- | --- | --- |
| Cause(s)  of death | tumor related | 780 (93.6) | 1175 (95.3) | 1955 (94.6) | 0.101 |
|  | - death only due to BM | 333 (25.4) | 447 (27.3) | 780 (26.5) |  |
|  | - death only due to extracranial metatases | 114 (8.7) | 239 (14.6) | 353 (12.0) |  |
|  | - death due to BM and extracranial metastases | 278 (21.2) | 425 (26.0) | 703 (23.8) |  |
|  | - unspecified | 55 (4.2) | 64 (3.9) | 119 (4.0) |  |
|  | not tumor related | 53 (6.4) | 58 (4.7) | 111 (5.4) |  |
|  | data missing | 478 | 404 | 882 |  |

Supplementary Table S2: Local BM therapy in HER-positive patients (supplementary material)

| **Local BM therapy modality** | **n (%)** |
| --- | --- |
| Only radiotherapy  - whole brain radiotherapy (WBRT)  - stereotactic radiotherapy (SRS)  - WBRT and SRS  - unknown | 758 (66%)  611 (81%)  83 (11%)  52 (7%)  12 (2%) |
| Only surgery | 61 (5%) |
| Surgery and radiotherapy  - WBRT  - SRS  - WBRT and SRS  - unknown | 325 (28%)  202 (62%)  70 (22%)  39 (12%)  14 (4%) |
