# Supplementary figures and images for: Characteristics of patients with brain metastases from human epidermal growth factor receptor 2-positive breast cancer: subanalysis of Brain Metastases in Breast Cancer Registry
Source: ESMO Open. 2022 May 30;7(3):100495. doi: 10.1016/j.esmoop.2022.100495 (PMC9271494; doi:10.1016/j.esmoop.2022.100495)

## Slide 1
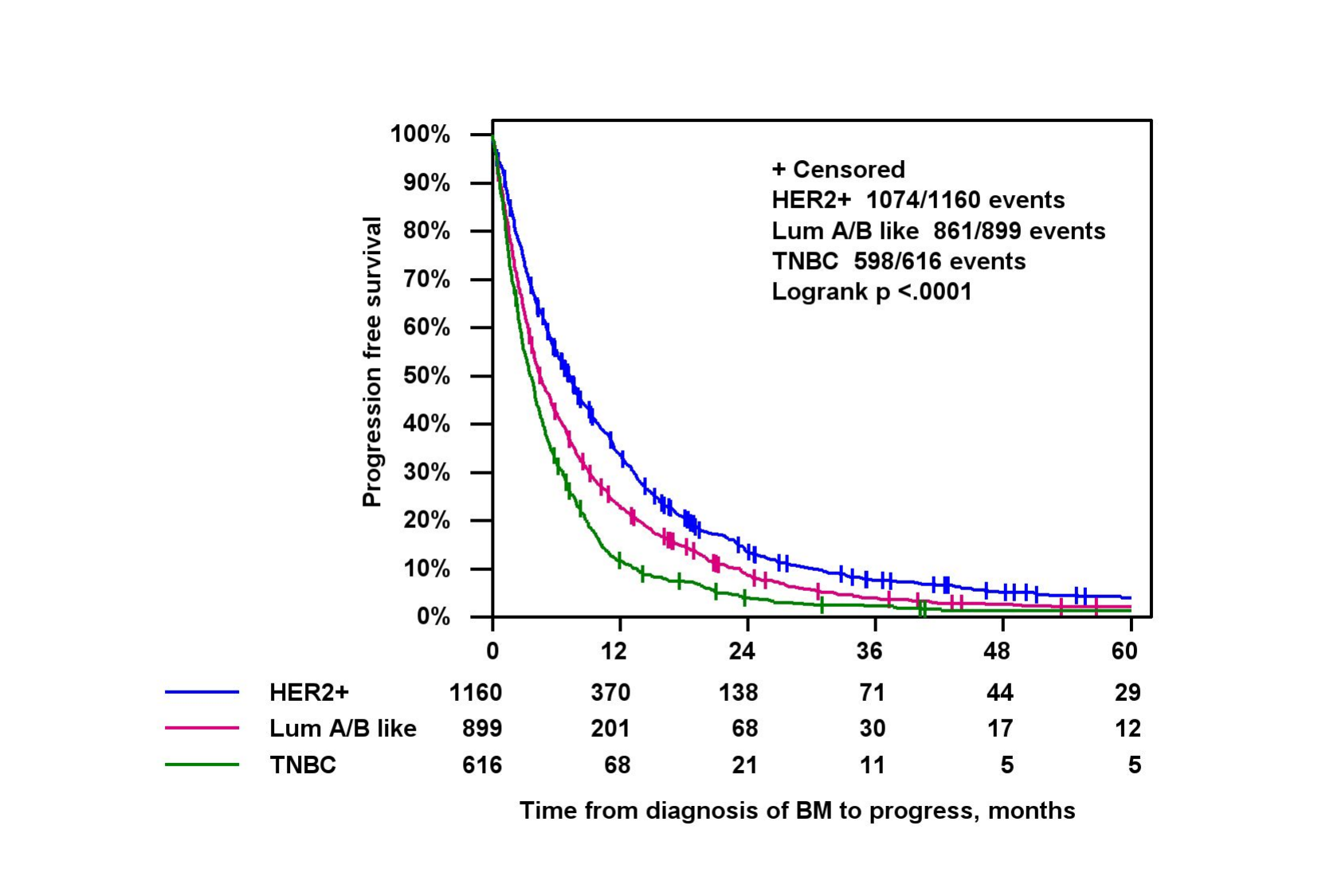

Supplement: Supplementary Figure S1 [file mmc2.pptx]

## Slide 1
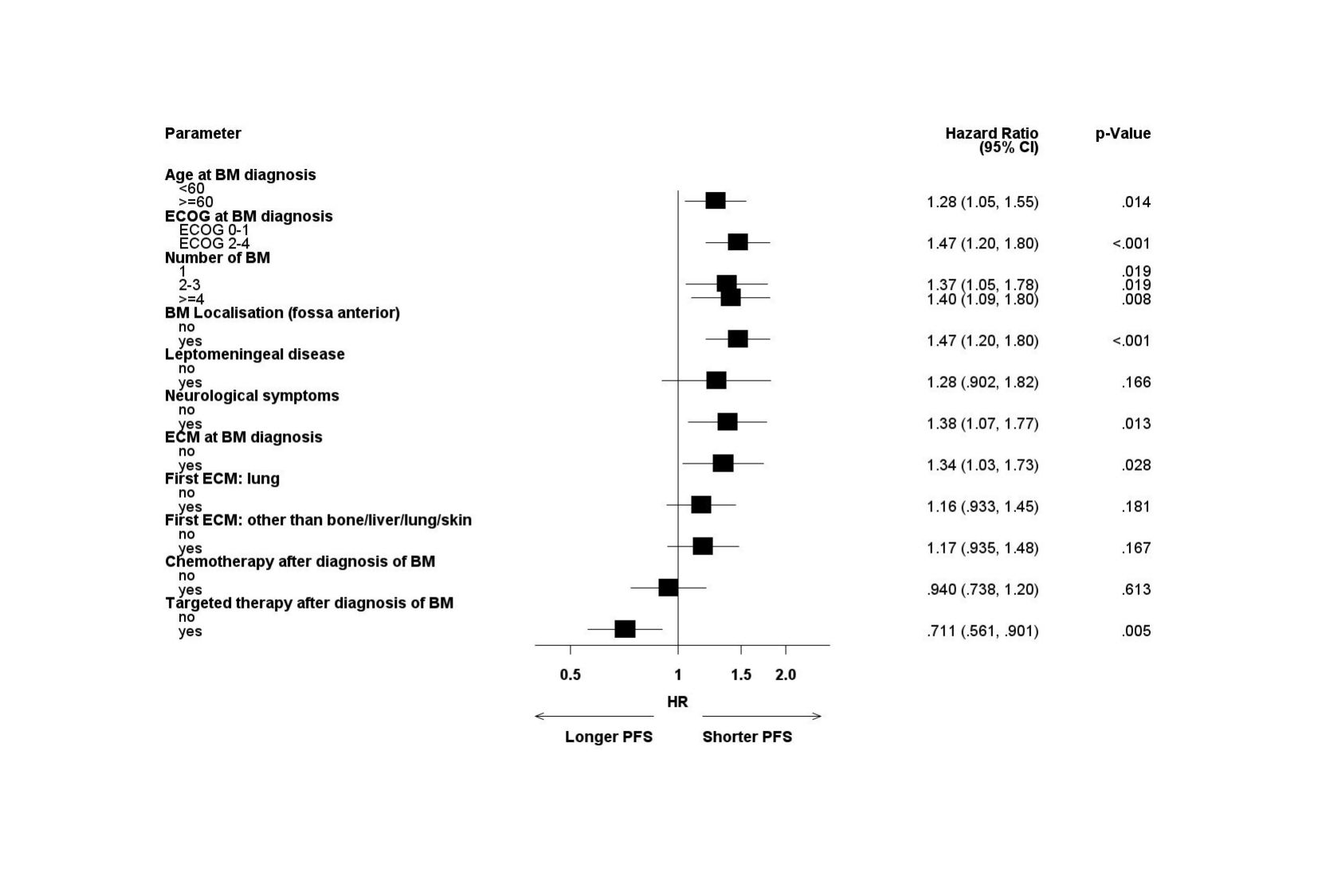

Supplement: Supplementary Figure S2 [file mmc3.pptx]

## Slide 1
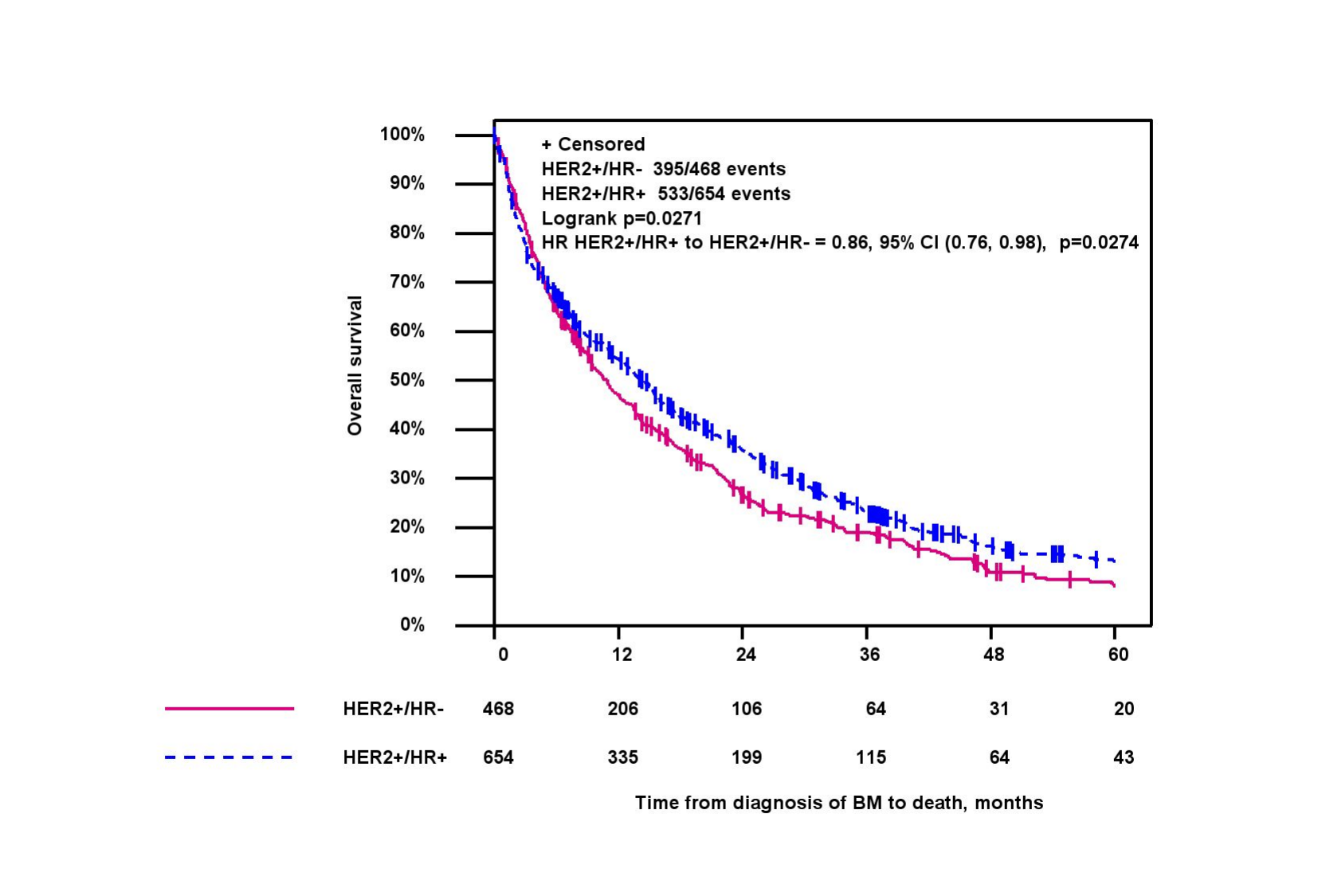

Supplement: Supplementary Figure S3 [file mmc4.pptx]

## Slide 1
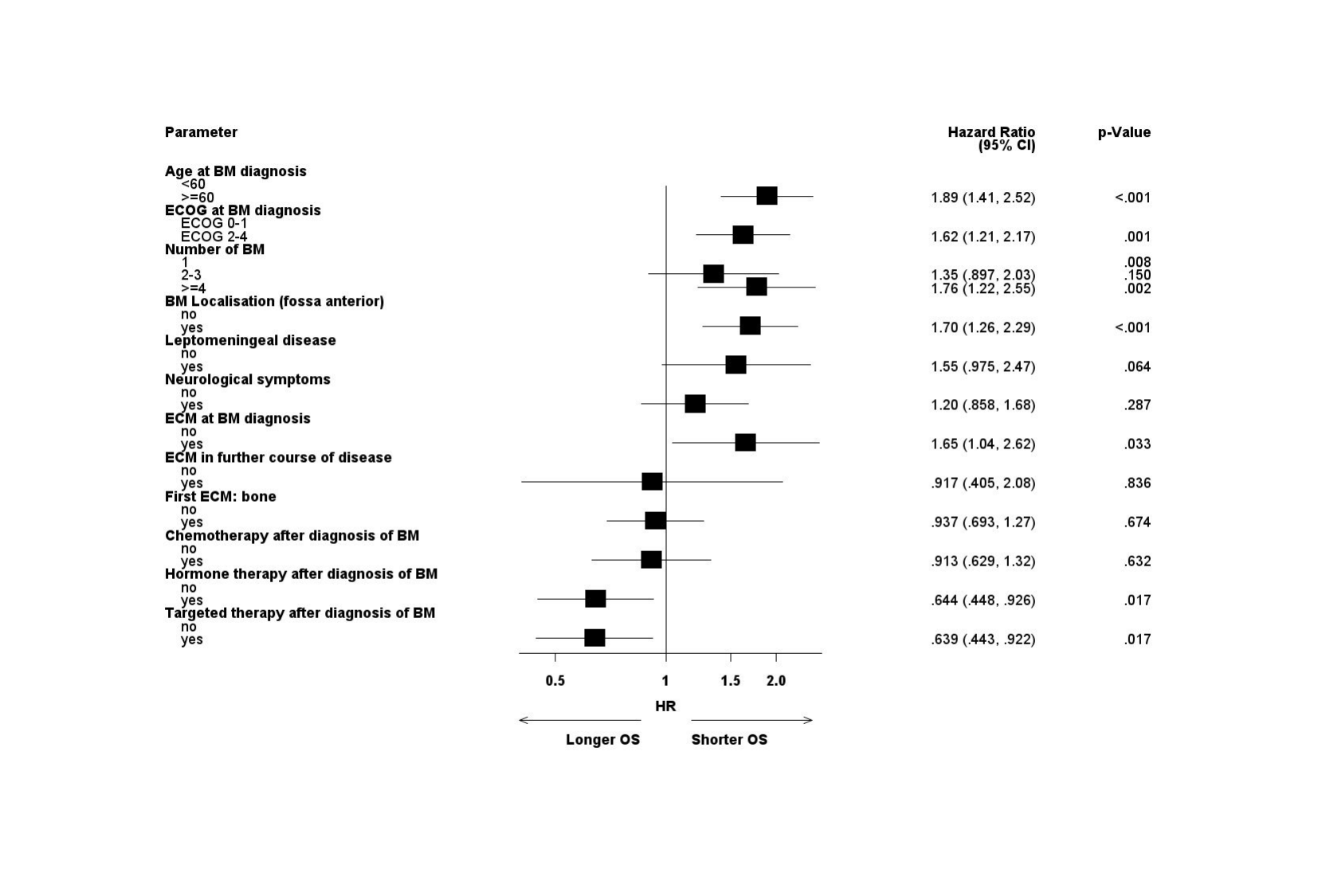

Supplement: Supplementary Figure S4 [file mmc5.pptx]
